# National Registry Data Analysis on a Unique Highly-Crosslinked Polyethylene for Total Hip Arthroplasty

**DOI:** 10.1016/j.artd.2023.101267

**Published:** 2023-11-18

**Authors:** Elda Paoli, Dario Bergadano, Shuya Sheng, Hemant Pandit

**Affiliations:** a Medacta International, Castel San Pietro, Switzerland; b University of Leeds, Leeds, UK; c Leeds Institute of Rheumatic and Musculoskeletal Medicine, University of Leeds, Leeds, UK

**Keywords:** Total hip arthroplasty, Highly-crosslinked polyethylene, HIGHCROSS, Registry

## Abstract

**Background:**

Several types of highly-crosslinked polyethylene with different manufacturing processes and mechanical properties are commercially available, including HIGHCROSS (Medacta). The aim of this registry study is to ascertain the long-term safety of the HIGHCROSS liners in total hip arthroplasty and compare the revision rates with its contemporaries using real-world data to establish their safety and generalizability when used by multiple surgeons.

**Methods:**

The Australian Orthopaedic Association National Joint Replacement Registry (AOANJRR) and the Swiss National Joint Registry (SIRIS) were selected for the large number of users of Medacta implants and the availability of long-term results. Three reports from SIRIS and 4 reports from AOANJRR were examined to establish the overall and Kaplan-Meier (KM) cumulative revision rate.

**Results:**

According to SIRIS, the HIGHCROSS liner was the most commonly used bearing surface with Medacta stems with overall revision rates for wear/osteolysis at 9.7 years of 0.04% and 0.03%, respectively, for AMIStem and Quadra. Based on AOANJRR, the KM cumulative revision rate for any reason of Medacta stems with HIGHCROSS liners was lower than that with ceramic liners at 3 years for MasterLoc and at 10 years for Quadra. The KM cumulative revision rate for any reasons of Medacta cementless cups with HIGHCROSS liners at 12 years was lower than the comparator made of all other implants and tribological couplings (1.6% vs 2.1%).

**Conclusions:**

This real-world data proves that long-term HIGHCROSS survival rates are comparable to other modern bearing surfaces.

## Introduction

Total hip arthroplasty (THA) is widely considered as one of the most successful surgical procedures in orthopaedics. It provides reproducible and reliable pain relief with recovery of the function and a significant improvement in quality of life following surgery. The first-generation hip prostheses used from 1960s to 2000 primarily used ultrahigh-molecular-weight polyethylene, also known as UHMWPE or conventional polyethylene (PE), as a bearing surface. These prostheses were successful in relieving pain and restoring mobility in less active and elderly patients. This success led to attempts to use the prostheses in more active and younger patients. This revealed increasing rates of revision primarily due to aseptic loosening after around 10 years’ implantation. The common mechanism of failure was the wear of the conventional PE leading to formation of particulate debris that induced osteolysis and contributed to implant loosening. The need for improved bearing surfaces was recognized, and thus conventional PE was progressively replaced by alternative materials such as highly cross-linked polyethylene (XLPE), ceramic, and metal. All 3 options were extensively studied by various research groups, engineers, and scientists and were introduced for clinical use in the late 1990s and early 2000s. Metal-on-metal bearings experienced a huge surge in their popularity in early 2000s, but equally dramatic drop in their usage due to the associated risks of adverse reaction to metal debris. At present, the most commonly used materials for the acetabular insert in THA are XLPEs and ceramics and different combinations are available to the surgeon when choosing the type of bearing couple: ceramic-on-ceramic, ceramic-on-XLPE, metal-on-XLPE. In the first decade since implantation, XLPE has demonstrated significant lower wear rates than conventional PE and therefore its popularity and clinical usage continue to increase. However, it is difficult to generalize this observation as a wide range of XLPEs are currently available on the market, and each is a different new material with unique mechanical properties, stability, and wear performance. The different types of XLPEs are characterized by: different types of radiation (gamma or electron beam) with varied dosages (between 50 and 100 kGy), different approaches to stabilization and recombination of free radicals (by annealing below the melt temperature or by re-melting above the melt temperature), and different sterilization methods (with gamma-rays or inert methods, such as ethylene oxide or gas plasma). High crosslinking improves the wear resistance of conventional PE but causes a slight decrease in other mechanical properties. For the same radiation level, these mechanical properties are strongly influenced by the crystallinity of the conventional PE, which can vary between 42% and 54%. Only careful control of the manufacturing process, particularly of the irradiation and cooling rate, provides the best compromise for XLPE between wear resistance and other mechanical properties. During radiation, crosslinking occurs, but there is also concomitant formation of free radicals. If, after radiation, the material is stabilized with heat treatment, free radicals can be eliminated. A significant reduction of free radicals is achieved only if the temperature rises over 135°C, which is the melting point of the conventional PE. This treatment brings the material to be oxidation-resistant, which in turn allows its mechanical properties to remain constant over time [1]. The different processes used to stabilize and cross-link the PE mean that the XLPE products available for routine clinical use can have very different properties and thereby impact their in-vivo performance. It is therefore important to establish mid- to long-term clinical outcomes in a large group of patients with the use of each of the currently available XLPE liners. National Joint Registries provide a unique opportunity to collate and analyze large datasets and, therefore, are an effective means of establishing safety and efficacy of a product. One commonly used XLPE is HIGHCROSS (Medacta International SA, Castel San Pietro, Switzerland). The HIGHCROSS liners are characterized by: radiation at 100 kGy, remelting temperature of 150°C, controlled proprietary cooling rate in order to optimize mechanical properties, and final sterilization with ethylene oxide. The aim of this study is to ascertain the long-term safety of the HIGHCROSSpolyethylene liners and compare the revision rates with its contemporaries by critically analyzing registry data.

## Material and methods

Two data sources were used in this analysis: the Australian Orthopaedic Association National Joint Replacement Registry (AOANJRR) [2] and the Swiss National Joint Registry (SIRIS) [3] were interrogated to analyze the long-term outcomes of the HIGHCROSS polyethylene bearing in THA in a large population. The AOANJRR was established in 1999 and the SIRIS in September 2012. These registries were chosen for 3 main reasons: the large number of users of Medacta implants and the availability of long-term results. Medacta started distribution of HIGHCROSS liners in Australia in 2008 and in Switzerland from the time of inception of SIRIS. In particular, 3 industry extended reports extracted from the SIRIS and 4 automated industry reports from the AOANJRR, focused on very popular Medacta hip implants, were analyzed. These reports provided information on the use and outcomes of the Medacta products compared to all other conventional THAs, based on data collected by the registries. The reports included all types of bearing couples, and the results are stratified by tribological pairing: metal-on-polyethylene (MoPE), metal-on-highly crosslinked polyethylene (MoXLPE), ceramic-on-polyethylene (CoPE), ceramic-on-highly crosslinked polyethylene (CoXLPE), and ceramic-on-ceramic (CoC). The HIGHCROSS liner’s safety was assessed through the revision rate on the total number of primary THAs and the cumulative revision rate according to the Kaplan-Meier method.

### Data extraction

**Figure 1:**
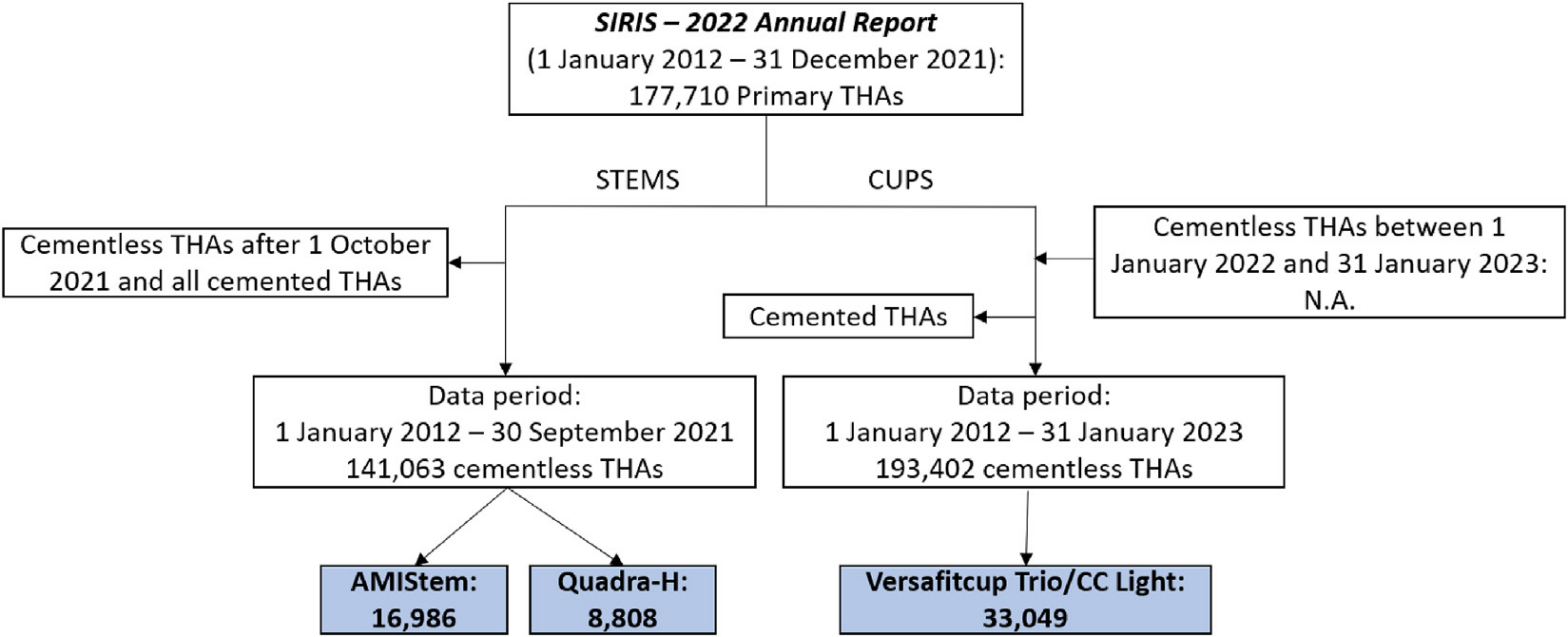
Flow chart of the data extraction for the SIRIS reports. If not available (N.A.), data will be available in the next SIRIS annual reports.

**Figure 2:**
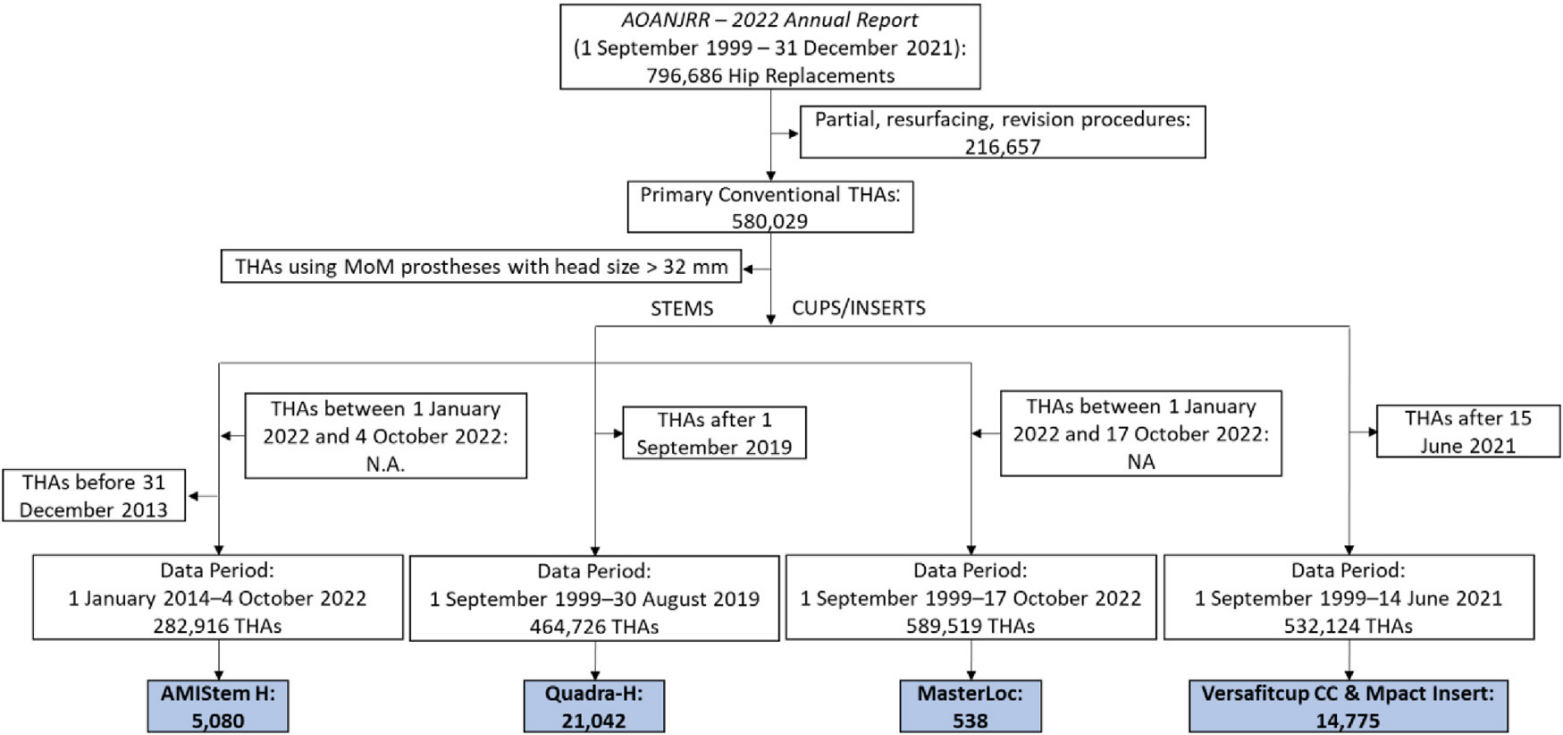
Flow chart of the data extraction for the AOANJRR reports. If not available (N.A.), data will be available in the next AOANJRR annual reports.

The data included in the reports was extracted from the joint replacement data recorded by the registries and referred to different time ranges depending on the specific implant considered. The flow charts of the data extraction, starting from the last available registry annual reports, are shown in Figure 1, Figure 2.

In the SIRIS Reports, the registrations are not always complete; individual components of multicomponent procedures may be missing, and therefore the total number of main product uses may not be the same as the sum of all derived mutually exclusive subgroups.

Since this study contains only a secondary analysis of deidentified aggregated data, the institutional review board approval is not applicable.

### Statistical methods

Statistical analyses were provided by SIRIS and AOANJRR, with no further analysis by the authors. This study relies on aggregated data only. Descriptive statistics are presented as absolute and relative frequencies, or mean and standard deviation. Survival rates and hazard ratios are reported with 95% confidence intervals (CIs). Statistical significance was determined based on the 95% CIs (*P* < .05).

## Results

In this section, the main results from the above-mentioned reports were reported separately.

### SIRIS - AMIStem uncemented hip stem (heads and inserts)

In the observational period, 16,986 primary THAs using AMIStem were performed in 15,365 patients by 283 surgeons in 66 different departments in Switzerland. The HIGHCROSS liner was used in 9241 cases (54.4%): 2735 cases with metal head and 6506 cases with ceramic head. Conventional PE insert was used in 724 cases (319 with metal head and 405 with ceramic head). In the remaining 6506 cases, CoC-bearing couple was used. The maximum follow-up was 9.7 years (mean: 4.3, SD: 2.6). 677 revision surgeries (3.9% of primary THAs) were reported. These included 13 cases (0.08% of primary THAs) that could be potentially attributed to wear and/or osteolysis: 6 were reported to manage issues associated with component wear, 2 for acetabular osteolysis, and 5 for femoral osteolysis. Seven of the 13 revisions (0.04% of primary THAs) involved the HIGHCROSS liner: 3 with the MoXLPE bearing and 4 with the CoXLPE. In the remaining 6 revisions, one involved the MoPE bearing, 3 the CoPE, and 2 the CoC. Complete data about the revisions are reported in Supplementary Figure 1.

The survival analysis of the implants showed that, focusing on the late revisions, which could be more likely related to the issue of PE wear, only 13 implants of 3080 still at risk at 7 years were later revised. Five of the 13 revisions involved the CoXLPE bearing and 5 the CoC, starting from a comparable number of implants for each bearing surface (6506 and 6250, respectively) and from a higher number of implants at risk at 7 years with the CoXLPE bearing against the CoC (1499 and 893, respectively) (Table 1). Only one revision involved the CoPE bearing, and no revision occurred with the HIGHCROSS liner coupled with metal head. More details are reported in Supplementary Figure 2.

**Table 1:** Survival of implants at 7 y.

Bearing surfaces	Revised <7 y	Lost to follow-up	Not yet reached 7 y	Still at risk at 7 y	Revised >7 y
MoPE	20	51	176	72	0
MoXLPE	143	195	2001	396	0
CoPE	23	58	104	220	1
CoXLPE	256	503	4248	1499	5
CoC	175	391	4791	893	5

### SIRIS - Quadra-H uncemented hip stem THA (heads and inserts)

In the observational period, 8808 primary THAs using Quadra-H stems were performed in 7949 patients by 194 surgeons in 56 different departments in Switzerland. The HIGHCROSS liner was used in 7039 cases (79.9%): 1845 cases with metal head and 5194 cases with ceramic head. The CoC bearing couple was used in 1411 cases, and the conventional PE insert was used in 136 cases (92 with metal head and 44 with ceramic head). The maximum follow-up was 9.7 years (mean: 3.8, SD: 2.4). 318 revision surgeries (3.6% of primary THAs) were performed, and 5 of them could be potentially attributed to wear. Three out of these 5 revisions (0.03% of primary THAs) involved the HIGHCROSS liner: 2 with CoXLPE bearing and one with the MoXLPE. In the remaining 2 revisions, one involved MoPE bearing and one involved CoPE. Complete data about the revisions are reported in Supplementary Figure 3.

The survival analysis of the implants still at risk at 7 years showed only 4 revisions performed after 7 years, all involving the HIGHCROSS liner (Table 2). This tribological bearing was used in the vast majority of the implants still at risk at 7 years (87%). More details are reported in Supplementary Figure 4.

**Table 2:** Survival of implants at 7 y.

Bearing surfaces	Revised <7 y	Lost to follow-up	Not yet reached 7 y	Still at risk at 7 y	Revised >7 y
MoPE	6	24	19	43	0
MoXLPE	83	182	1169	411	3
CoPE	3	6	14	21	0
CoXLPE	182	445	4049	518	1
CoC	24	94	1213	80	0

### SIRIS – Versafitcup Trio/CC light cups (THA)

In the observational period, 33,049 primary THAs using Versafitcup acetabular cups (in particular, Versafitcup Trio and Versafitcup CC series) were performed in 29,547 patients by 356 surgeons in 75 different departments in Switzerland. These cups were coupled in 21,738 cases (65.8%) with a HIGHCROSS liner, in 10,096 cases (30.6%) with a ceramic liner, and in 904 cases (2.7%) with conventional PE. The maximum follow-up was 10 years (mean: 1.9, SD: 2.4). The cumulative percent revision (CPR) at 10 years of any component of Versafitcup with HIGHCROSS liner was 7.0%, against the 14.7% of Versafitcup with conventional PE liner, and it was comparable to that of all other cementless cups with ceramic liner (7.0% and 6.4%, respectively) (Table 3). More details were reported in Supplementary Figure 5.

**Table 3:** Yearly cumulative percent revision (CPR) of primary THAs by bearing surface (any component revision).

CPR	1 y	3 y	5 y	7 y	10 y
Versafitcup + XLPE	2.4 (2.2, 2.6)	3.5 (3.2, 3.7)	4.5 (4.2, 4.8)	5.6 (5.2, 6.0)	7.0 (6.4, 7.6)
Versafitcup + PE	4.2 (3.0, 5.7)	4.7 (3.5, 6.3)	6.3 (4.8, 8.3)	9.8 (7.6, 12.7)	14.7 (10.3, 20.7)
Other cementless cups + C	2.6 (2.4, 2.9)	3.9 (3.6, 4.2)	4.6 (4.3, 5.0)	5.1 (4.8, 5.5)	6.4 (5.8, 7.1)

### AOANJRR – Automated Industry Report 1561 - Quadra-H total conventional hip

In this case 21,042 THAs with Quadra-H were performed in 18,353 patients by 183 surgeons in 164 different hospitals in Australia from June 2007 to August 2019. The maximum follow-up was 12.2 years (mean: 4.46, SD: 2.64). In this case 674 revision surgeries were performed; 4 of them (0.02% of primary THAs) were for osteolysis, while no cases of wear of acetabular insert or wear of the acetabulum occurred. Focusing on the bearing surfaces, the CPR with the HIGHCROSS liner was comparable with that with the ceramic insert. In particular, the CPR at 10 years after surgery was 5.6%, 5.3%, and 5.1% for CoC, CoXLPE, and MoXLPE bearings, respectively (Table 4). More details were reported in Supplementary Figure 6.

**Table 4:** Yearly cumulative percent revision (CPR) of Quadra-H primary THAs by bearing surface.

CPR	1 y	3 y	5 y	10 y
CoC	1.4 (1.2, 1.6)	2.3 (2.1, 2.6)	3.0 (2.7, 3.3)	5.6 (4.6, 6.7)
CoXLPE	2.2 (1.8, 2.7)	3.2 (2.7, 3.8)	3.9 (3.3, 4.6)	5.3 (4.3, 6.6)
MoXLPE	2.3 (1.8, 3.0)	3.1 (2.5, 3.9)	3.8 (3.1, 4.8)	5.1 (4.0, 6.4)

### AOANJRR – Automated Industry Report 5307 - AMIStem H total conventional hip

**Figure 3:**
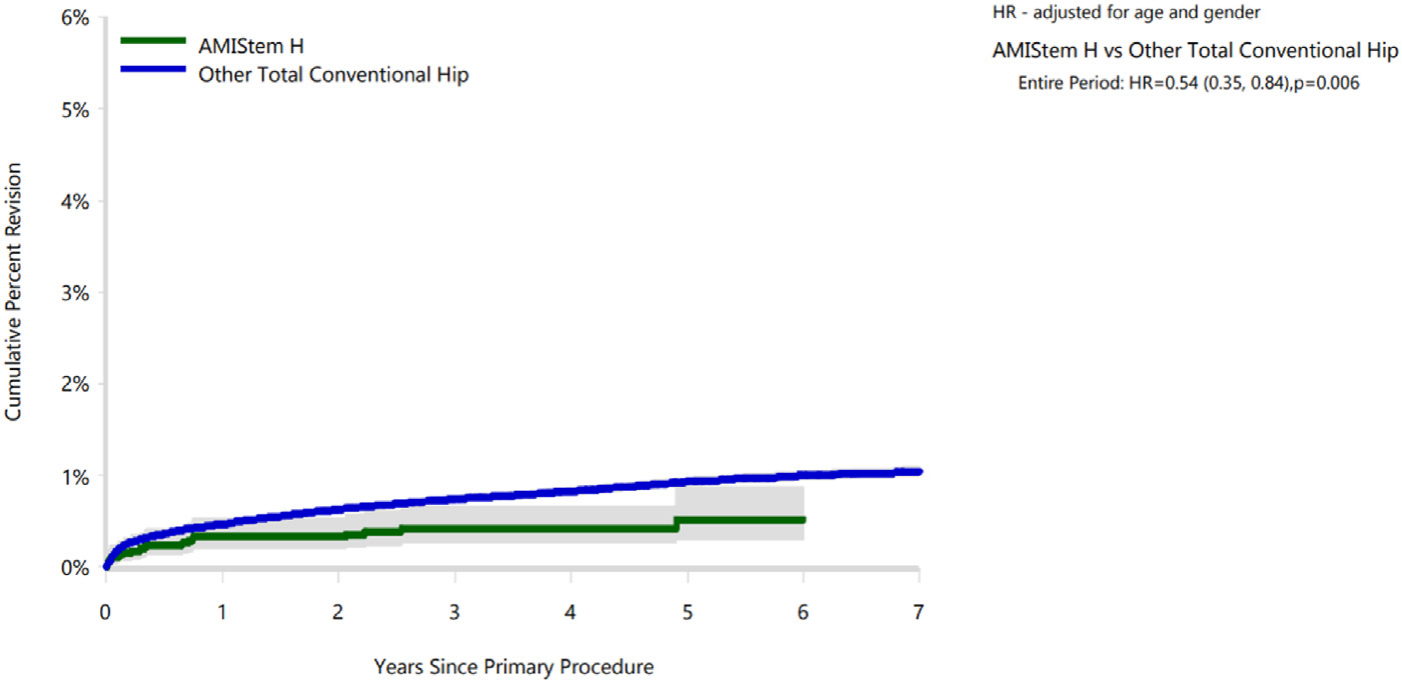
Cumulative percent revision (CPR) of primary THAs by model (acetabular component revision).

In this case 5080 primary THAs with AMIStem-H were performed in 4500 patients by 61 surgeons in 78 different hospitals in Australia from March 2014 to September 2021. The maximum follow-up was 7.54 years (mean: 3.16, SD: 1.81). In this case 110 revision surgeries in the AMIStem-H group were performed, and none of the revisions were attributed to osteolysis, wear of acetabular insert, or wear of the acetabulum. Focusing on the revision of the acetabular components, at 6 years of follow-up, the CPR of the AMIStem-H group (0.5%) was half that of all other THAs, both cementless and cemented (1.0%) (Table 5, Fig. 3). More details are reported in Supplementary Figure 7.

**Table 5:** Yearly cumulative percent revision (CPR) of primary THAs by model (acetabular component revision).

CPR	1 y	3 y	5 y	6 y
AMIStem	0.3 (0.2, 0.5)	0.4 (0.3, 0.7)	0.5 (0.3, 0.9)	0.5 (0.3, 0.9)
Other THAs	0.5 (0.4, 0.5)	0.7 (0.7, 0.8)	0.9 (0.9, 1.0)	1.0 (1.0, 1.0)

### AOANJRR – Automated Industry Report 8221 - MasterLoc total conventional hip

In this case 538 primary THAs with MasterLoc were performed in 515 patients by 21 surgeons in 33 different hospitals in Australia from March 2016 to August 2022. The maximum follow-up was 6.57 years (mean: 3.48, SD: 1.74). In this case 9 revision surgeries were performed: 4 (0.7% of primary THAs) involved the CoC bearing and 5 (0.9% of primary THAs) the CoXLPE. None of these revisions were attributed to osteolysis, wear of acetabular insert, or wear of the acetabulum. The CPR of the CoXLPE bearing at 5 years of follow-up (2.1%) was lower than the one of the CoC bearing at 3 years (2.4%) (Table 6). More details are reported in Supplementary Figure 8.

**Table 6:** Yearly cumulative percent revision (CPR) of MasterLoc primary THAs by bearing surface.

CPR	1 y	2 y	3 y	5 y
CoXLPE	1.5 (0.6, 4.1)	1.5 (0.6, 4.1)	1.5 (0.6, 4.1)	2.1 (0.9. 5.0)
CoC	1.7 (0.5, 5.3)	2.4 (0.9, 6.3)	2.4 (0.9, 6.3)	-

### AOANJRR – Automated Industry Report 4413 - Versafitcup CC & Mpact total conventional HIP

**Figure 4:**
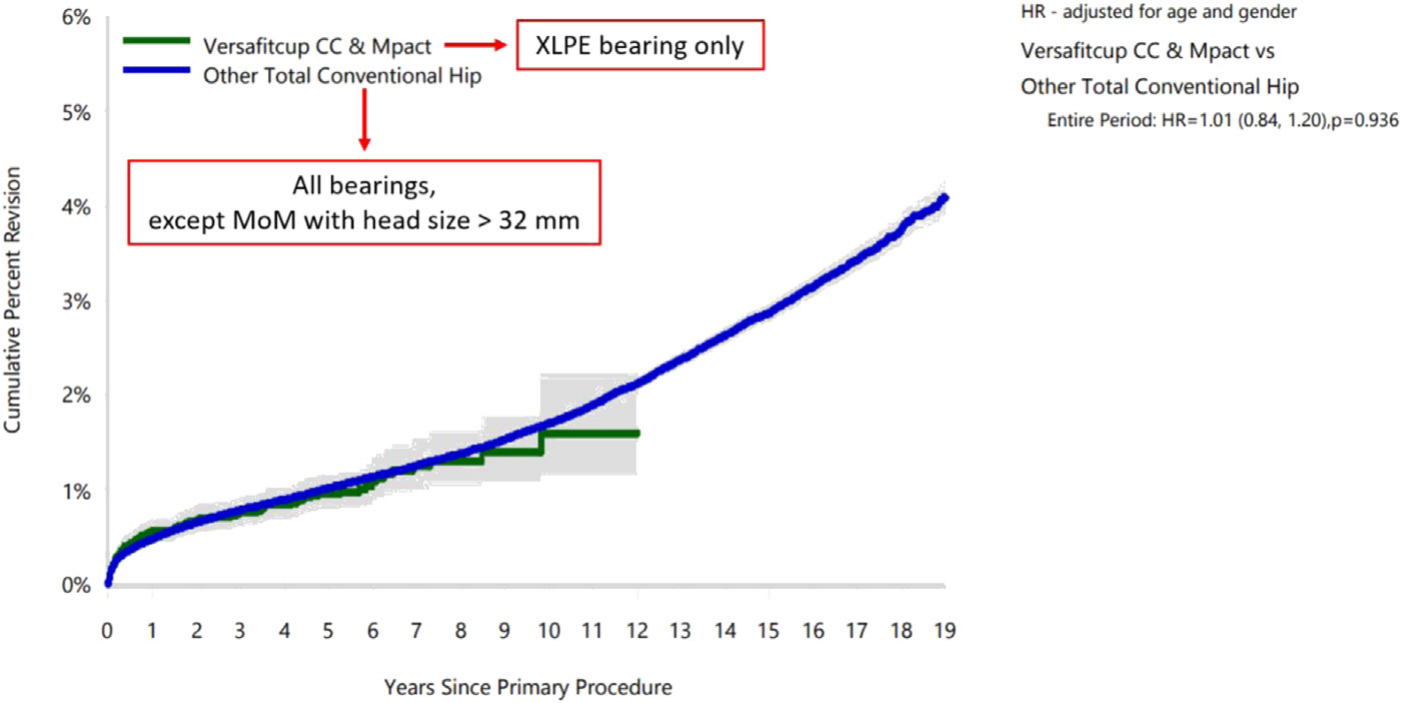
Cumulative percent revision (CPR) of primary THAs by model (acetabular component revision).

This report included primary procedures involving the HIGHCROSS liner used either with Versafitcup CC series or Mpact acetabular cups. In this case 14,775 primary THAs were performed in 13,351 patients by 197 surgeons in 171 different hospitals in Australia from July 2008 to May 2021. The maximum follow-up was 12.9 years (mean: 3.86, SD: 2.73). In this case 465 revision surgeries were performed: indication for revision was listed as osteolysis in 3 cases (0.02% of primary THAs), while no case of wear of acetabular insert occurred. In the comparison group, composed by 517,349 other THAs, both cementless and cemented, including CoC bearing and excluding metal-on-metal couplings larger than 32 mm, the osteolysis caused 350 revisions, which represented 0.07% of the primary surgeries. The percentages of the 2 groups were comparable. Focusing on the revision of the acetabular components, at 12 years after surgery the CPR of Versafitcup CC and Mpact with the HIGHCROSS liner (1.6%) was lower than the one of the other THAs (2.1%) (Table 7, Fig. 4). More details are reported in Supplementary Figure 9.

**Table 7:** Yearly cumulative percent revision (CPR) of primary THAs by model (acetabular component revision).

CPR	1 y	3 y	5 y	8 y	10 y	12 y
Versafitcup CC & Mpact (XLPE bearing only)	0.6 (0.4, 0.7)	0.8 (0.6, 0.9)	1.0 (0.8, 1.2)	1.3 (1.0, 1.6)	1.6 (1.1, 2.2)	1.6 (1.1, 2.2)
Other THAs (All bearings except metal-on-metal with head size >32 mm)	0.5 (0.5, 0.5)	0.8 (0.8, 0.8)	1.0 (1.0, 1.0)	1.4 (1.3, 1.4)	1.7 (1.7, 1.7)	2.1 (2.1, 2.2)

## Discussion

This study confirms the long-term safety of the HIGHCROSS liners when used in primary THA, based on the reports extracted from the Australian and Swiss Registries. SIRIS Reports on AMIStem and Quadra stems showed that the HIGHCROSS liner was the most used tribological partner (54.4% and 79.9%, respectively). The revision rates for wear and osteolysis at 9.7 years of these Medacta stems coupled with the HIGHCROSS liners were very low: 0.04% and 0.03%, respectively, for AMIStem and Quadra. SIRIS Report on Versafitcup acetabular cups confirmed a significant improvement of the performance of the HIGHCROSS against the conventional PE, despite the possible differences in the 2 groups. The Kaplan-Meier cumulative revision rate of HIGHCROSS liner at 10 years (7.0%, CI: 6.4-7.6) was not statistically superior to the rate of all other cementless cups with ceramic liner (6.4%, CI: 5.8-7.1). AOANJRR Reports on Medacta stems coupled with HIGHCROSS liners showed that the CPR for any reason of the femoral component was lower than that with ceramic liners for MasterLoc at 3 years (CoXLPE: 1.5%; CoC: 2.4%) and for Quadra at 10 years (CoXLPE: 5.3%; MoXLPE: 5.1%; CoC: 5.6%). AOANJRR Report on Medacta cementless cups coupled with HIGHCROSS liners indicated that the CPR for any reason of the acetabular component at 12 years was lower than the comparator made of all other implants and tribological couplings (1.6% vs 2.1%) including ceramic-on-ceramic bearings. It is worth pointing out that the revision of the femoral component almost always involves the replacement of the PE liner, while this doesn’t occur with the ceramic liner, and this could affect the results.

To our knowledge, few registry studies were performed focusing on the survival rates of the XLPE in THA and none specifically on HIGHCROSS. In the registry study performed by Davis et al. [4], the superiority of the XLPE over the conventional PE was proved analyzing the effect of the PE irradiation on the survival of cemented and reverse hybrid THAs. Based on data from the National Joint Registry of England, Wales, Northern Ireland, and the Isle of Man, linked with manufacturing data supplied by manufacturers, they found that the XLPE has been associated with a significant reduction in the risk of revision for aseptic loosening against the conventional PE. This finding is not in total agreement with the results of the analysis of the Nordic Arthroplasty Register Association database performed by Johanson et al. [5] to determine whether the use of XLPE would mean a reduced risk of revision. The Kaplan-Meier survival analysis revealed non-significant differences between XLPE and conventional PE in the risk of revision, both for any reason and for aseptic loosening for the cemented cups. It is possible that these findings could be attributed to the use of cemented cups in the elderly/less active population. For the cementless cups, a significantly lower survival was noted in the conventional PE group for all-cause revision but not for aseptic loosening. We are not able to provide logical explanation for this finding. Sharplin et al. [6] investigated the revision rates of all bearings for primary THAs registered on the New Zealand Joint Registry to determine which bearing coupling has been the most successful and durable over the last 16 years. Their findings indicated that the CoXLPE bearing had the lowest revision rate for any reason (0.54/100 component-years), followed by CoC (0.61/100 component-years) and MoXLPE (0.61/100 component-years).

There are many strengths in this study. The large number of patients and the use of implants by surgeons from multiple centers confirm the safety and efficacy of HIGHCROSS, and therefore the results can be considered as true representation of real-world data. In addition, the large number of analyzed data allowed us to reduce the influence of the outliers and confounders on the results. The data collection and analysis were carried out by independent members and included consecutive cases without exclusion of those in the learning curve of an individual surgeon. This registry study has some limitations. First, the maximum follow-up available was 12 years after surgery, and each report referred to a different follow-up period. Second, it was not a randomized controlled clinical trial, which is the gold standard for ascertaining the efficacy and safety of a treatment. Third, the indication and the need for revision are strongly surgeon- and country-dependent. Fourth, patient and surgeon factors can’t be differentiated from those implant-related. Further investigations over a longer period should be performed to confirm the long-term safety of the HIGHCROSS, also including additional information such as patient demographics and preoperative diagnosis. In addition, a matched follow-up period should be considered to have comparable results.

## Conclusions

This registry study was conducted to analyze the long-term outcomes of the HIGHCROSS polyethylene bearing in THA. The data extracted from the Australian and Swiss registries proved that the HIGHCROSS polyethylene produced by Medacta International SA allows survival rates comparable to the main alternative products on the market and that the life-time limit of the devices manufactured with this material has not yet been reached.

## Conflicts of interest

Paid employee for a company or supplier: Medacta International (E. Paoli, D. Bergadano). Paid consultant for a company or supplier: Zimmer Biomet, Allay Therapeutics, Medacta International, Paradigm Pharma, Smith and Nephew, MAT Ortho, Teleflex, Invibio (H. Pandit). Stock or stock options in a company or supplier: Medacta International (D. Bergadano). Research support from a company or supplier as a principal investigator: Zimmer Biomet, Depuy Synthes, Invibio (H. Pandit). Medical/Orthopedic publications editorial/governing board: Editor, Journal of Arthroscopy and Joint Surgery (H. Pandit).

For full disclosure statements refer to https://doi.org/10.1016/j.artd.2023.101267.

## Appendix A Supplementary data

Conflict of Interest Statement for All Authors
