# Supplementary material for: National Registry Data Analysis on a Unique Highly-Crosslinked Polyethylene for Total Hip Arthroplasty
Source: Arthroplast Today. 2023 Nov 18;24:101267. doi: 10.1016/j.artd.2023.101267 (PMC10679760; doi:10.1016/j.artd.2023.101267)
Supplement: Conflict of Interest Statement for All Authors [file mmc1.pdf]

CONFLICT OF INTEREST STATEMENT

American Association of Hip and Knee Surgeons

(Adopted from the American Academy of Orthopaedic Surgeons disclosure statement)

The following form **must be filled out completely and submitted by each author (example, 6 authors, 6 forms). All items require a response. If there is no relevant disclosure for a given item, enter "*None.*"**

National registry data analysis on a unique highly-crosslinked polyethylene for total hip arthroplasty

1. Royalties from a company or supplier (The following conflicts were disclosed) None

2. Speakers bureau/paid presentations for a company or supplier (The following conflicts were disclosed) None

3A. Paid employee for a company or supplier (The following conflicts were disclosed)
Medacta International

3B. Paid consultant for a company or supplier (The following conflicts were disclosed) None

3C. Unpaid consultants for a company or supplier (The following conflicts were disclosed) None

4. Stock or stock options in a company or supplier (The following conflicts were disclosed) None

5. Research support from a company or supplier as a Principal Investigator (The following conflicts were disclosed) None

6. Other financial or material support from a company or supplier (The following conflicts were disclosed) None

7. Royalties, financial or material support from publishers (The following conflicts were disclosed) None

8. Medical/Orthopaedic publications editorial/governing board (The following conflicts were disclosed) None

9. Board member/committee appointments for a society (The following conflicts were disclosed) None

Each author must sign AND print or type his/her name, date and submit a separate form

In addition, one BLINDED Conflict of Interest form (no author names used) should be submitted per manuscript with all author disclosures.

Elda Paoli	Elda Paoli	22/06/2023
Author Name (Print or Type)	Author Signature	Date

CONFLICT OF INTEREST STATEMENT

American Association of Hip and Knee Surgeons

(Adopted from the American Academy of Orthopaedic Surgeons disclosure statement)

The following form **must be filled out completely and submitted by each author (example, 6 authors, 6 forms). All items require a response. If there is no relevant disclosure for a given item, enter "*None*."**

National registry data analysis on a unique highly-crosslinked polyethylene for total hip arthroplasty

1. Royalties from a company or supplier (The following conflicts were disclosed) none

2. Speakers bureau/paid presentations for a company or supplier (The following conflicts were disclosed) none

3A. Paid employee for a company or supplier (The following conflicts were disclosed) Employed by Medacta Int.l

3B. Paid consultant for a company or supplier (The following conflicts were disclosed) none

3C. Unpaid consultants for a company or supplier (The following conflicts were disclosed) none

4. Stock or stock options in a company or supplier (The following conflicts were disclosed) Medacta Int.l

5. Research support from company or supplier as a Principal Investigator (The following conflicts were disclosed) none

6. Other financial or material support from a company or supplier (The following conflicts were disclosed) none

7. Royalties, financial or material support from publishers (The following conflicts were disclosed) none

8. Medical/Orthopaedic publications editorial/governing board (The following conflicts were disclosed) none

9. Board member/committee appointments for a society (The following conflicts were disclosed) none

Each author must sign AND print or type his/her name, date and submit a separate form

In addition, one BLINDED Conflict of Interest form (no author names used) should be submitted per manuscript with all author disclosures.

DARIO BERGADANO	[signature]	27/06/2023
Author Name (Print or Type)	Author Signature	Date

CONFLICT OF INTEREST STATEMENT

American Association of Hip and Knee Surgeons

(Adopted from the American Academy of Orthopaedic Surgeons disclosure statement)

The following form **must be filled out completely and submitted by each author (example, 6 authors, 6 forms). All items require a response. If there is no relevant disclosure for a given item, enter "*None.*"**

National registry data analysis on a unique highly-crosslinked polyethylene for total hip arthroplasty

1. Royalties from a company or supplier (The following conflicts were disclosed)
None

2. Speakers bureau/paid presentations for a company or supplier (The following conflicts were disclosed)
None

3A. Paid employee for a company or supplier (The following conflicts were disclosed)
None

3B. Paid consultant for a company or supplier (The following conflicts were disclosed)
None

3C. Unpaid consultants for a company or supplier (The following conflicts were disclosed)
None

4. Stock or stock options in a company or supplier (The following conflicts were disclosed)
None

5. Research support from a company or supplier as a Principal Investigator (The following conflicts were disclosed)
None

6. Other financial or material support from a company or supplier (The following conflicts were disclosed)
None

7. Royalties, financial or material support from publishers (The following conflicts were disclosed)
None

8. Medical/Orthopaedic publications editorial/governing board (The following conflicts were disclosed)
None

9. Board member/committee appointments for a society (The following conflicts were disclosed)
None

Each author must sign AND print or type his/her name, date and submit a separate form

In addition, one BLINDED Conflict of Interest form (no author names used) should be submitted per manuscript with all author disclosures.

Author Name (Print or Type): Shuya Sheng Author Signature Shuya Sheng Date: 22/06/2023

CONFLICT OF INTEREST STATEMENT

American Association of Hip and Knee Surgeons

(Adopted from the American Academy of Orthopaedic Surgeons disclosure statement)

The following form **must be filled out completely and submitted by each author (example, 6 authors, 6 forms). All items require a response. If there is no relevant disclosure for a given item, enter "*None*."**

National registry data analysis on a unique highly-crosslinked polyethylene for total hip arthroplasty

1. Royalties from a company or supplier (The following conflicts were disclosed) None

2. Speakers bureau/paid presentations for a company or supplier (The following conflicts were disclosed) None

3A. Paid employee for a company or supplier (The following conflicts were disclosed) None

3B. Paid consultant for a company or supplier (The following conflicts were disclosed)
Zimmer Biomet, Allay Therapeutics, Medacta International, Paradigm Pharma, Smith and Nephew, MAT Ortho, Teleflex, Invibio

3C. Unpaid consultants for a company or supplier (The following conflicts were disclosed) None

4. Stock or stock options in a company or supplier (The following conflicts were disclosed) None

5. Research support from a company or supplier as a Principal Investigator (The following conflicts were disclosed)
Zimmer Biomet, Depuy Synthes, Invibio

6. Other financial or material support from a company or supplier (The following conflicts were disclosed) None

7. Royalties, financial or material support from publishers (The following conflicts were disclosed) None

8. Medical/Orthopaedic publications editorial/governing board (The following conflicts were disclosed)
Editor Journal of Arthroscopy and Joint Surgery

9. Board member/committee appointments for a society (The following conflicts were disclosed) None

Each author must sign AND print or type his/her name, date and submit a separate form

In addition, one BLINDED Conflict of Interest form (no author names used) should be submitted per manuscript with all author disclosures.

Hemant Pandit		21st June 2023
Author Name (Print or Type)	Author Signature	Date
