# Supplementary material for: National Registry Data Analysis on a Unique Highly-Crosslinked Polyethylene for Total Hip Arthroplasty
Source: Arthroplast Today. 2023 Nov 18;24:101267. doi: 10.1016/j.artd.2023.101267 (PMC10679760; doi:10.1016/j.artd.2023.101267)
Supplement: Supplementary Material [file mmc2.docx]

### *SIRIS - AMIStem uncemented hip stem (heads and inserts)*


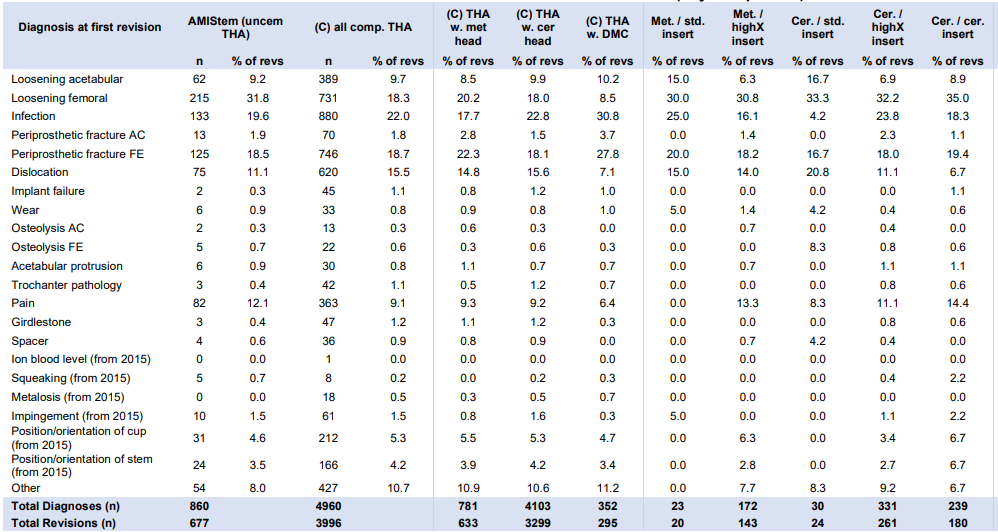


Figure 1: Clinical characteristics at first revision (any component)


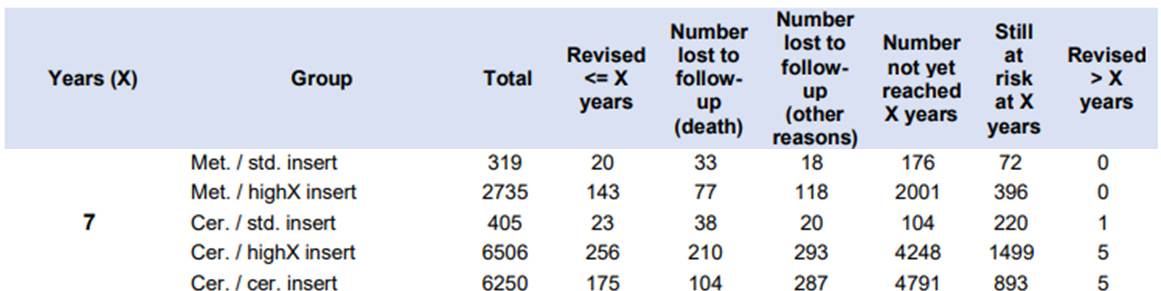


Figure 2: Survival of implants at 7 years (subgroups)

### *SIRIS - Quadra-H uncemented hip stem THA (heads and inserts)*


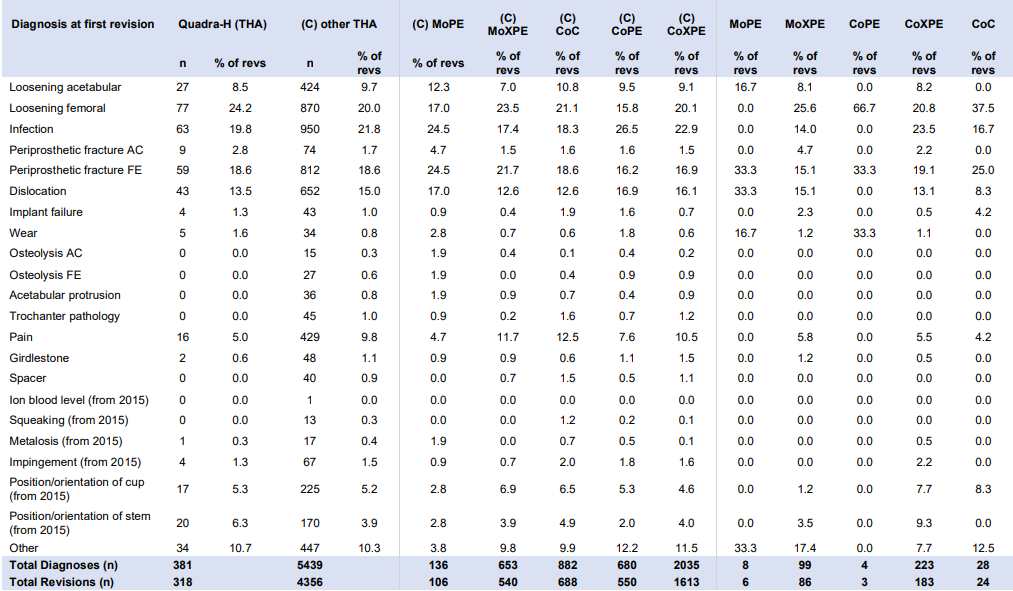


Figure 3: Clinical characteristics at first revision (any component)


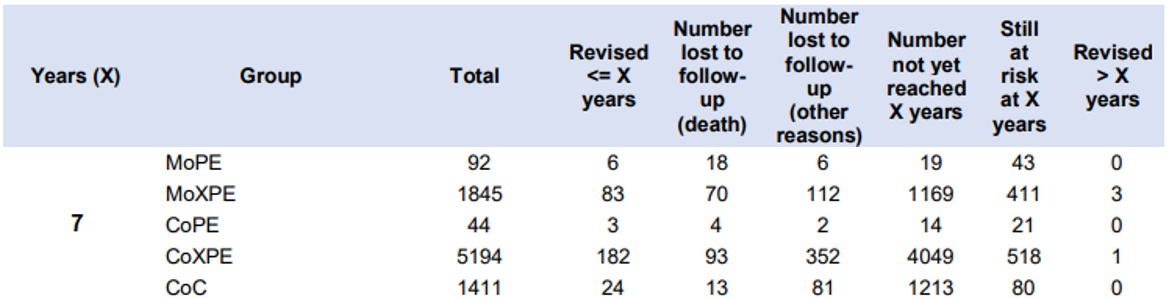


Figure 4: Survival of implants at 7 years (subgroups)

### *SIRIS – Versafitcup Trio/CC light cups (THA)*


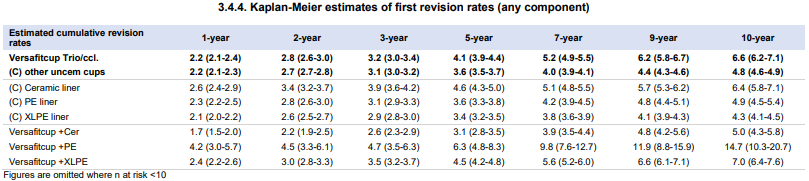


Figure 5: Kaplan-Meier estimates of first revision rates (any component)

### *AOANJRR – Automated Industry Report 1561 - Quadra-H Total Conventional Hip*


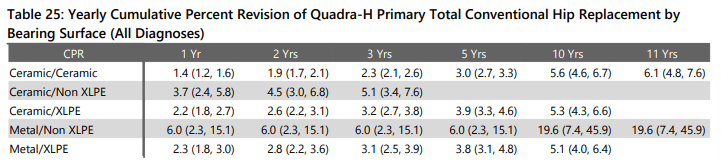


Figure 6: Yearly Cumulative Percent Revision of Quadra-H Primary Total Conventional Hip Replacement by Bearing Surface (All Diagnoses)

### *AOANJRR – Automated Industry Report 5307 - AMIStem H Total Conventional Hip*


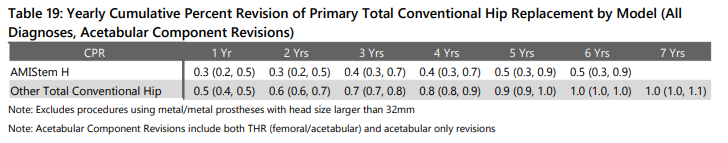


Figure 7: Yearly Cumulative Percent Revision of Primary Total Conventional Hip Replacement by Model (All Diagnoses, Acetabular Component Revisions)

### *AOANJRR – Automated Industry Report 8221 - MasterLoc Total Conventional Hip*


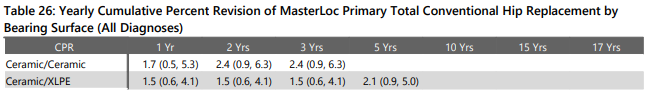


Figure 8: Yearly Cumulative Percent Revision of MasterLoc Primary Total Conventional Hip Replacement by Bearing Surface (All Diagnoses)

### *AOANJRR – Automated Industry Report 4413 - Versafitcup CC & Mpact Total Conventional HIP*


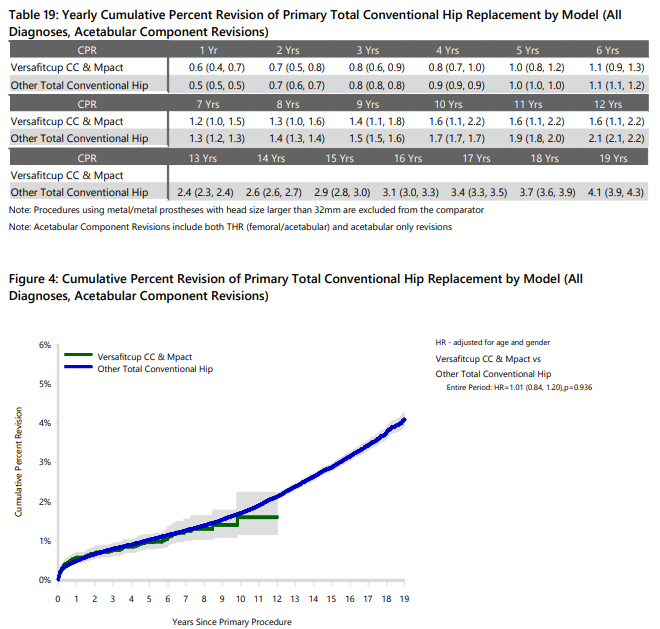


Figure 9: Yearly Cumulative Percent Revision of Primary Total Conventional Hip Replacement by Model (All Diagnoses, Acetabular Component Revisions
